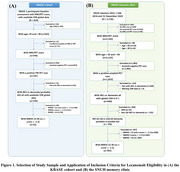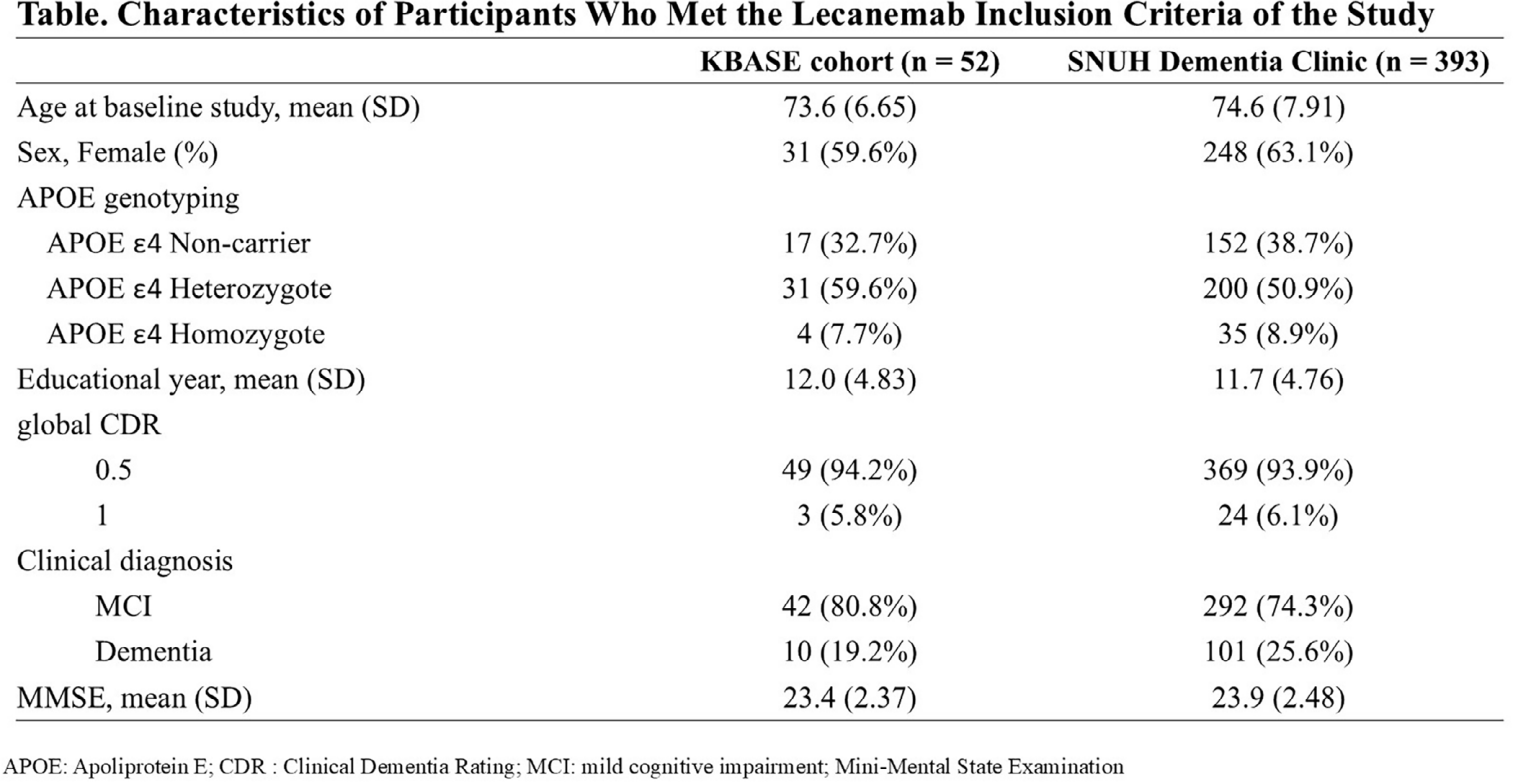# Lecanemab Eligibility in Korean Memory Clinic and Research Populations: A Retrospective Analysis

**DOI:** 10.1002/alz70860_106483

**Published:** 2025-12-23

**Authors:** So Yeon Jeon, Min Soo Byun, Hyeji Choi, Yoon Hee Kim, Chung Hee Gwag, Gijung Jung, Dong Young Lee

**Affiliations:** ^1^ Chungnam National University Hospital, Daejeon, Daejeon, Korea, Republic of (South); ^2^ School of Medicine, Chungnam National University, Daejeon, Korea, Republic of (South); ^3^ Seoul National University Hospital, Seoul, Korea, Republic of (South); ^4^ Department of Neuropsychiatry, Seoul National University Hospital, Seoul, Korea, Republic of (South); ^5^ Seoul National University Medical Research Center, Seoul, Korea, Republic of (South); ^6^ Seoul National University Hosipital, Seoul, Korea, Republic of (South); ^7^ Department of Neuropsychiatry, Seoul National University Hospital, Seoul, Korea, Republic of (South); ^8^ Seoul National University Dementia Research Center, Seoul, Korea, Republic of (South)

## Abstract

**Background:**

Despite lecanemab's approval in South Korea in May 2024, limited information is available for the proportion of patients who are eligible for lecanemab treatment in Korea. This study aims to estimate the proportion of lecanemab treatment eligible patients among individuals who visited a university memory clinic (clinical population) and individuals who participated a cohort study for Alzheimer's disease (AD) (research population) in Korea.

**Methods:**

This retrospective study included 2,726 individuals who visited the Memory Clinic at Seoul National University Hospital (SNUH) between January 2019 and November 2024, presenting with cognitive decline, and underwent standard clinical evaluations. Additionally, 625 participants from the Korean Brain Aging Study for the Early Diagnosis and Prediction of Alzheimer's Disease (KBASE) cohort who completed similar clinical evaluations at baseline were included. The ADRD‐TWG Appropriate Use Recommendations (AUR) were used as the lecanemab eligibility criteria. Regarding MMSE score criterion, we applied not only 22 or higher, but z‐scores greater than ‐1.5.

**Results:**

Among the clinical sample (*N* = 2,726) 1,053 individuals (38.6%) were identified as amyloid PET positive. After applying additional clinical criteria, 740 participants remained eligible, accounting for 27.2% of the initial sample. Finally, applying MMSE criterion further reduced the eligible group to 393 participants (14.4%) of the initial sample. In the cohort sample (*N* = 625), after applying amyloid PET positivity and cognitive impairment criteria, 150 participants (24%) remained eligible. Further applying MMSE criterion led to a final eligible population of 52 participants (8.3%) (Figure 1). The characteristics of participants meeting the inclusion criteria are summarized in Table 1. Among the clinical sample, 28 participants (7.1%) had more than four microbleeds on brain MRI, 14(3.6%) had superficial siderosis, 62(15.8%) had two or more lacunar infarcts, and 37(9.4%) had cortical infarcts. Severe WMH was observed in six participants (19.3%). Ultimately, 237 participants were deemed eligible for lecanemab treatment, representing 8.7% of the initial sample.

**Conclusion:**

This study provides the first real‐world analysis of lecanemab eligibility in Korean population. The findings highlight the substantial proportion of early AD patients may not qualify under current guidelines. These results underscore the need for tailored eligibility criteria that reflect population‐specific characteristics and healthcare accessibility in South Korea.